# Systematic description of COVID-19 pandemic using exact SIR solutions and Gumbel distributions

**DOI:** 10.1007/s11071-022-07907-4

**Published:** 2022-09-29

**Authors:** J. E. Amaro

**Affiliations:** Departamento de Física Atómica, Molecular y Nuclear and Instituto Carlos I de Física Teórica y Computacional, Universidad de Granada, 18071 Granada, Spain

**Keywords:** COVID-19 coronavirus, SIR model, Differential equations, Gumbel distribution

## Abstract

An epidemiological study is carried out in several countries analyzing the first wave of the COVID-19 pandemic using the SIR model and Gumbel distribution. The equations of the SIR model are solved exactly using the proper time as a parameter. The physical time is obtained by integration of the inverse of the infected function over proper time. Some properties of the solutions of the SIR model are studied such as time scaling and the asymmetry, which allows to obtain the basic reproduction number from the data. Approximations to the solutions of the SIR model are studied using Gumbel distributions by least squares fit or by adjusting the maximum of the infected function. Finally, the parameters of the SIR model and the Gumbel function are extracted from the death data and compared for the different countries. It is found that ten of the selected countries are very well described by the solutions of the SIR model, with a basic reproduction number between 3 and 8.

## Introduction

Since the declaration of the COVID-19 pandemic by the World Health Organization in March 2020, studies by the mathematical epidemiologist community have intensified and models of various kinds have been developed in order to provide insights and make predictions about the spread of the disease [1–4]. Epidemiological models have been used as basic tools in epidemiology for over a century [5–13] and have been extensively used prior to the COVID-19 pandemic [14–17].

A wide range of models have been proposed and tested in an attempt to describe the COVID-19 data and forecast the future evolution of the pandemic in different regions of the planet. From very simple models [18, 19] to numerous variants of compartmental models based on the SIR model (susceptible, infected, removed) have been proposed [20–23]. Additional models have been employed such as the SEIR model [24], which adds the exposed compartment of individuals, the uncertain SIR model [25], and others that include different parameters that statistically describe the many factors that may influence the pandemic dynamics.

It is worth mentioning the SITR model [26], which also includes the treatment process T, and the SITRS model [27], which includes the possibility that recovered people can lose their temporary immunity against the virus and subsequently join the susceptible compartment again. These models may also include two or more susceptible groups of individuals $S_1, S_2$,... to take into account the different susceptibilities due to age or other factors. Disease simulations are obtained by solving a system of nonlinear differential equations, or by means of discretization methods of different types, such as the discrete fractional model [27].

More sophisticated approaches also take into account the spatial spread of the disease, not just the temporal one. Models may involve partial differential equations in space and time coordinates, or, alternatively, stochastic methods [28–31]. Nonetheless, it has been argued that complex models with numerous parameters may not necessarily be advantageous without having enough data for a meaningful validation [32]. More recent studies on the mathematical modeling of the COVID-19 pandemic can be found in the Refs. [33–39].

After going through the sixth wave in many countries, the world data [40] show that a comprehensive description of the entire time series appears to be an impossible task, since each country presents its own characteristics (e.g., diverse lock-down and social-economic measures). Therefore, in this work we proceed by studying the first wave for those countries that present data with a similar structure and that can unambiguously be described mathematically using an epidemiological model of the SIR type.

The reason for using the SIR model specifically in this work is because it is the simplest possible model to describe the evolution of an infectious disease. In our case we intend to describe mortality regardless of the detailed description of the intermediate compartments through which individuals may pass. One of the objectives is to determine which countries, if any, can be described with the SIR model, to obtain its parameters and, finally, to compare between the countries to see if some kind of universality of the model can be observed.

By inspection, from the recorded worldometer data [40] we found that there are only nine or ten such countries (we leave aside the case of China that has been exhaustively studied and where the pandemic apparently died out without the need for vaccines). Among those countries there are eight Europeans—Spain, France, Italy, UK, Germany, Belgium, Switzerland and Sweden—together with Canada and USA. Moreover, we have also added the cases of India and Brazil. In this work we will carry out a systematic study of the pandemic in each of them by studying the series of cumulative deaths and daily deaths. Our hypothesis is that deaths, *D*(*t*), can be considered a fraction of removals, *R*(*t*), both cumulative and daily, and therefore, both functions follow an epidemiological curve that will differ essentially in a normalization constant and a shift in time.

Our purpose is to investigate if data can be described with simple epidemiological curves using the SIR model and the even simpler Gumbel function [41]. A fundamental question about the first wave of COVID-19 is whether the lockdown limitations had an effect in reducing the number of deaths. Non-pharmaceutical interventions (NPI) are still under debate. A recent meta-analysis review [42] fails to confirm that lock-downs have had a large, significant effect on mortality rates. If the daily mortality curves fit well with a basic SIR model, it would be interesting to conclude affirmatively or negatively regarding the effect of NPI on them.

The structure of the paper is as follows. In Sect. 2 we review the solutions of the SIR model that will be considered here. We describe in detail how to obtain numerical solutions as a function of the proper time, depending on the parameters $\beta$ and the basic reproduction number $\rho ={\mathcal R_0}=\lambda /\beta$. In Sect. 3 We examine how well the Gumbel function fits exact SIR solutions with only one parameter, barring normalization and a temporal shift. In Sect. 4 we will discuss the time scaling of SIR solutions and define an asymmetry parameter that depends linearly on $\rho$ and therefore can be used to characterize the value of the basic reproduction number from a set of data. In Sect. 5 we present our results of fits of death data with the exact SIR model and Gumbel functions. In Sect. 6 we draw our conclusions.

## Solutions of the SIR model and the proper time

In this section we briefly describe the SIR model and discuss its analytical solution in terms of, what we will call here, *proper time*, $\tau$, which is a natural variable to measure time through the proportion of removals, where the SIR equations have a trivial and easily interpreted solution. The real time is then obtained by integrating the exact solution.

In the SIR model the individuals of a closed population *N* affected by a contagious disease are divided into three types: susceptible, *S*, infected, *I*, and removed (recovered or dead), *R*. As functions of time, the number of individuals in each compartment is assumed to verify the following equations

1 $$\begin{aligned}&\frac{\mathrm {d}{} \textit{S}}{\mathrm {d}{} \textit{t}} = - \frac{\lambda }{N}IS , \end{aligned}$$

2 $$\begin{aligned}&\frac{\mathrm {d}{} \textit{R}}{\mathrm {d}{} \textit{t}} = \beta I , \end{aligned}$$

3 $$\begin{aligned}&I = N -R -S. \end{aligned}$$

In this transmission-dynamics system the first equation means that the variation of susceptible individuals decreases by infection and is proportional to the number of susceptible and the number of infected individuals. The constant $\lambda$ measures the rate of infection. The second equation describes the removal variation as proportional to the number of infected individuals, the removal rate being $\beta$. By the third equation, the difference between the total number minus the susceptible individuals minus the removed ones must be the number of infected at each instant *t*.

We will consider the initial values $S (0) = S_{_0} <N$ and $R (0) = 0$. Therefore, $I (0) = N-S_{_0}> 0$. There must be a number, albeit small, of infected in the system initially for the epidemic to begin. For convenience below we will work with the percentages of susceptible, infected and recovered individuals, over the total number of the population, which are obtained by dividing by *N*:

4 $$\begin{aligned} s(t)= \frac{S}{N}, i(t)= \frac{I}{N}, r(t)= \frac{R}{N}, \end{aligned}$$

with $s(0)=s_{_0}$, $i(0)=1-s_{_0}$, and $r(0)=0$.

The proper time, $\tau$, is defined as the temporal variable that describes naturally the evolution of the epidemic, by counting the evolution of the recovered individuals, *r*(*t*), which is always an increasing function with time. From the second SIR equation in differential form, it is defined by

5 $$\begin{aligned} \mathrm{d}R = \beta I \mathrm{d}t \equiv N \mathrm{d}\tau , \end{aligned}$$

So, the definition of proper time is $\mathrm{d}\tau = \mathrm{d}r$ and the interpretation of this variable is that we measure the change in time using the change in recoveries as the biological clock instead of using the physical clock. If we demand that $\tau = 0$ for $t = 0$, we trivially have

6 $$\begin{aligned} \tau = r(t). \end{aligned}$$

The idea of proper time is based on other equivalent approaches described, e.g., in [43, 44], where the susceptible function *s* is used as variable instead of *r*. The proper time is nothing more than a change of the time variable into a more convenient one. In our case, time is measured by counting the number of recovered (in percent), since *r*(*t*) is an increasing function, although it does not depend linearly on time. Note that the recovered function verify $0\le R \le N$ and therefore $0 \le r(t) \le 1$. Thus, by definition the proper time has a range limited by

7 $$\begin{aligned} 0 \le \tau \le 1. \end{aligned}$$

From Eq. (5) we have

8 $$\begin{aligned} I = \frac{N}{\beta }\frac{\mathrm{d}\tau }{\mathrm{d}{} \textit{t}}, i = \frac{1}{\beta }\frac{\mathrm{d}\tau }{\mathrm{d}{} \textit{t}}. \end{aligned}$$

Thus, the change of the physical time is given by

9 $$\begin{aligned} \mathrm{d}t = \frac{1}{\beta }\frac{\mathrm{d}\tau }{i(\tau )}, \end{aligned}$$

where $i(\tau )$ are the infected percent expressed as a function of proper time.

To obtain the susceptible function note that we can write, inserting Eq. (5) into Eq. (1)

10 $$\begin{aligned} \mathrm{d}S = - \frac{\lambda }{N}IS \mathrm{d}t = - \frac{\lambda }{\beta } S \mathrm{d}\tau = -\rho S \mathrm{d}\tau , \end{aligned}$$

where $\rho$ is the so-called basic reproduction number

11 $$\begin{aligned} \rho \equiv {{\mathcal {R}}}_0 \equiv \frac{\lambda }{\beta }. \end{aligned}$$

The parameter $\rho$ has here the meaning of being the decay constant of the susceptible population in units of proper time. Equation (10) is readily integrated giving

12 $$\begin{aligned} S = S_{_0}\text {e}^{-\rho \tau }, \end{aligned}$$

Thus, *S* follows an exponential decay law as a function of proper time. The constant

13 $$\begin{aligned} \tau _{1/2} = \frac{ \ln 2}{ \rho } = \frac{ \beta \ln 2 }{ \lambda } \end{aligned}$$

represents the half-life in proper time units, i.e., the length of proper time after which the susceptible population is reduced to half.

**Fig. 1:**
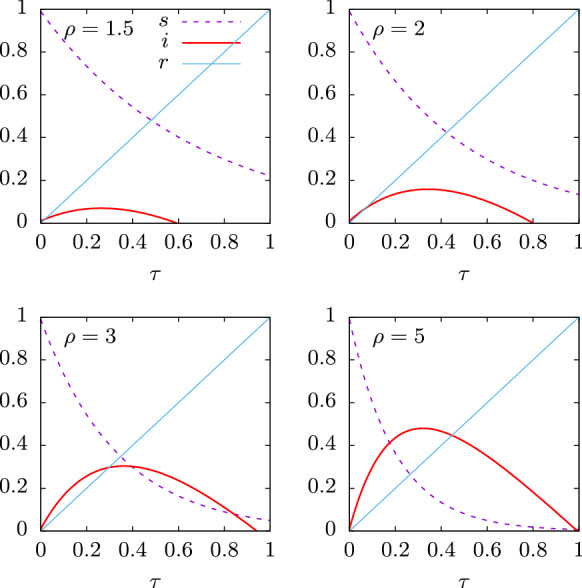
Solution of the SIR equations as a function of proper time $\tau$ for initial susceptible $s_{_0}=0.99$ and for several values of the basic reproduction number $\rho ={{\mathcal {R}}}_0$

Finally, the third SIR equation (3) gives directly the infected population as a function of proper time

14 $$\begin{aligned}&I = N -N\tau - S_{_0}\text {e}^{-\rho \tau } \end{aligned}$$

15 $$\begin{aligned}&i(\tau ) = 1 -\tau - s_{_0}\text {e}^{-\rho \tau }, \end{aligned}$$

with the condition $0 \le i \le 1$. For $\tau =0$ we obtain the initial number of infected population $i_{_0}=1-s_{_0}$. The end of the epidemic is reached when $i = 0$. This happens for a value of the proper time $\tau = \tau _f >0$ that is the solution of the transcendental equation

16 $$\begin{aligned} i(\tau _f) = 0 \Longrightarrow \tau _f + s_{_0}\text {e}^{-\rho \tau _f} = 1 \end{aligned}$$

In Fig. 1 we show some numerical examples. The analytical solutions of the SIR equations are plotted as a function of proper time for various values of the basic reproduction number $\rho =1.5,$ 2, 3 and 5. In all cases we assume that $s_{_0} = 0.99$; i.e., that one percent of the population is initially infected.

**Fig. 2:**
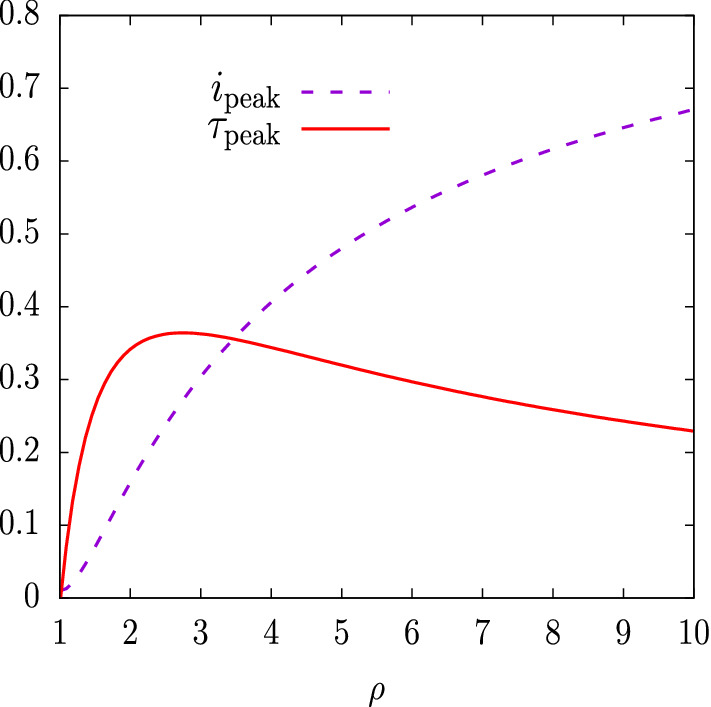
The peak values of *i*, $\tau$ as a function of the basic reproduction number $\rho$. For $s_{_0}=0.99$ the peak values are almost independent of $s_{_0}$

In Fig. 1 we see that the number of infected individuals as a function of $\tau$ first grows to a maximum and then decreases to zero. The maximum of $i(\tau )$ is the peak of the epidemic. It is reached for $\mathrm{d}i/\mathrm{d}\tau =0$. Then

17 $$\begin{aligned} -1+\rho s_{_0}\text {e}^{-\rho \tau } = 0, \end{aligned}$$

and therefore

18 $$\begin{aligned} s_{_0}\text {e}^{-\rho \tau } = \frac{1}{\rho }. \end{aligned}$$

From here the peak of the epidemic verifies

19 $$\begin{aligned}&\tau _{\mathrm{peak}} = \frac{\ln s_{_0}\rho }{\rho } \end{aligned}$$

20 $$\begin{aligned}&s_{\mathrm{peak}}= s(\tau _{\mathrm{peak}}) = \frac{1}{\rho } \end{aligned}$$

21 $$\begin{aligned}&i_{\mathrm{peak}}=i(\tau _{\mathrm{peak}}) = 1- \frac{1+\ln s_{_0}\rho }{\rho }. \end{aligned}$$

A condition for the existence of this maximum for $\tau _{\mathrm{peak}}>0$, from Eq. (19), is that $s_{_0} \rho > 1$. If we assume that, at the very beginning of the epidemic, $s_{_0}$ is very close to 1, then it is enough that $\rho > 1$ for the epidemic to begin to grow [45]. In that case, $i(\tau )$ starts growing up to a maximum reached at $\tau _{\mathrm{peak}}$, where it starts to decrease to zero.

In Fig. 2 we show the peak values of the infected rate $i_\text {peak}(\rho )$ in the epidemic as a function of the basic reproduction number $\rho$, for $s_{_0} = 0.99$. Since $s_{_0}$ is very close to one and the dependence on $s_{_0}$ is logarithmic, these peak values are almost independent on the precise value of $s_{_0} \simeq 1$. The peak value of infected individuals grows with the basic reproduction number $\rho$. When $\rho$ is very large, above 10, the logarithmic dependence on the numerator makes the peak to grow more slowly. For $\rho = 10$ we have $i_\text {peak} = 0.67$, that is, at the peak of the epidemic two thirds of the population will be infected simultaneously. For $\rho =50$, more than 90% of the population will be infected simultaneously at the peak.

In Fig. 2 we also show the value of the proper time at the epidemic peak, $\tau _\text {peak}(\rho )$. It presents a maximum for

22 $$\begin{aligned}&\frac{\mathrm{d}\tau _{\text {peak}}}{d\rho }=\frac{1-\ln s_{_0}\rho }{\rho ^2}=0 \end{aligned}$$

23 $$\begin{aligned}&\ln s_{_0}\rho = 1 \Longrightarrow \rho = \frac{e}{s_{_0}} \simeq 2.72, \end{aligned}$$

for $s_{_0} \simeq 1$. The maximum value of $\tau _\text {peak}$ is then

24 $$\begin{aligned} (\tau _\text {peak})_\text {max}= \frac{s_{_0}}{e} \simeq 0.367 \end{aligned}$$

for $\rho = e = 2.72$.

**Fig. 3:**
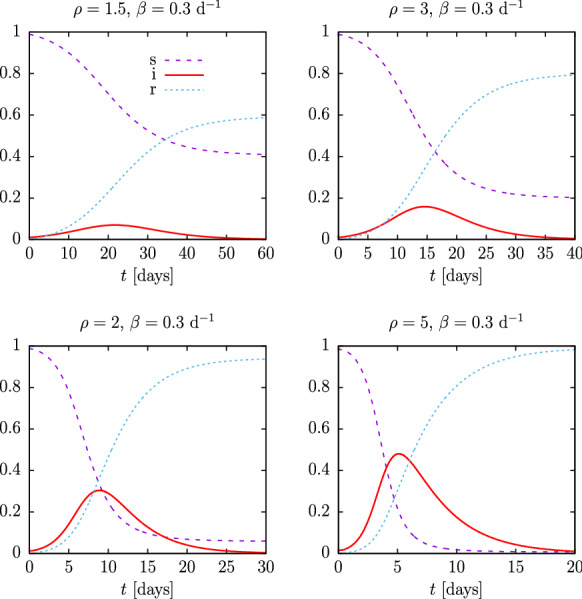
Solution of the SIR equations as a function of physical time, *t*, for initial susceptible $s_{_0}=0.99$, for $\beta =0.3\,\hbox {d}^{-1}$, and for several values of the reproduction number $\rho ={{\mathcal {R}}}_0$

Finally, starting from the analytical solution of the SIR equations as a function of the proper time, $i(\tau )$, we will proceed to obtain the solution as a function of the physical time, *t*. It is obtained from Eq. (9) by integrating between 0 and $\tau$. Assuming that $t = 0$ for $\tau = 0$, we obtain

25 $$\begin{aligned} t(\tau ) = \frac{1}{\beta }\int _0^\tau \frac{\mathrm{d}\tau }{i(\tau )} = \frac{1}{\beta }\int _0^\tau \frac{\mathrm{d}\tau }{1-\tau -s_{_0}\text {e}^{-\rho \tau }} \end{aligned}$$

This integral is not analytical, but it can be calculated numerically with precision using any numerical algorithm, such as Simpson’s rule or Gaussian integration, since the function inside the integral is quite smooth.

The final SIR solution is then obtained in parametric form by tabulating $t (\tau )$, $i (\tau )$, $\tau$ and $s (\tau )$. By plotting $i (\tau )$, $\tau$ and $s (\tau )$ as a function of $t (\tau )$ we obtain the results of Fig. 3, corresponding to the exact (numerical) solution of the SIR equations as a function of physical time, for the same parameters of Fig. 1 and for $\beta =0.3$ d$^{-1}$.

Note in Fig. 3, firstly, that the height of the maximum of *i*(*t*) coincides with that of the maximum of the analytical solution $i(\tau )$ of Fig. 1, as it should be, since we have only made a change of variable—from the proper time to the physical time. Of course, the dependence on physical time has drastically changed. For a constant recovery rate $\beta =0.3$ d$^{-1}$, by increasing the value of the basic reproduction number, $\rho$, the epidemic passes more quickly and lasts less time, almost explosively for $\rho = 5$—for which it lasts only 20 days—compared to $\rho = 1.5$—where it lasts almost two months.

Note that the time in Eq. (25) is inversely proportional to $\beta$. Making $\beta$ smaller, time becomes larger and with it the duration of the epidemic. This will be seen in more detail in Sect. 3.1 when we discuss the time scaling property.

Therefore, assuming that the recovery rate, $\beta$, in an epidemic is somewhat constant, the basic reproduction number $\rho$ largely determines the evolution of the pandemic. The analytical solution allows estimating the maximum number of simultaneous infections at the peak, $i_\text {peak}$, from this number. This does not depend much on $s_{_0}$, as long as this number is close to one, due to its dependence on the logarithm of $s_o\rho$.

**Fig. 4:**
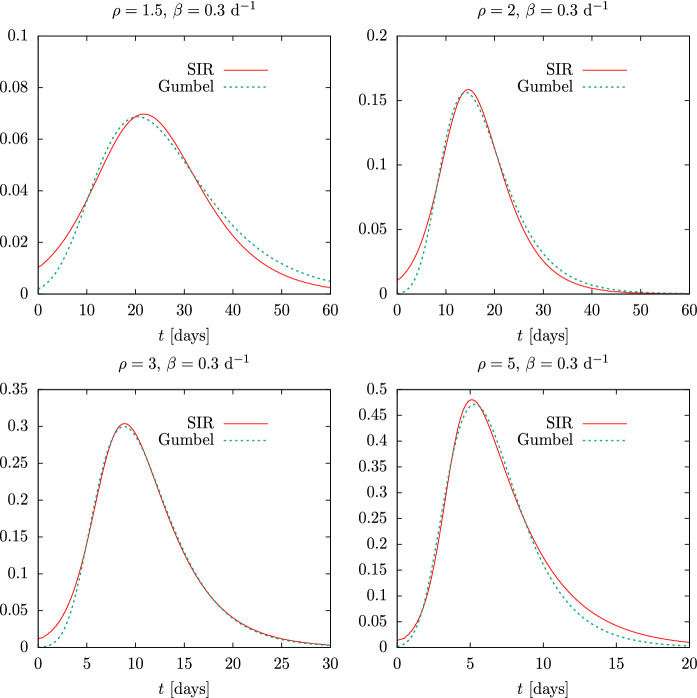
Gumbel distribution compared to the exact Solution of the SIR equations for $s_{_0}=0.99$, $\beta =0.3\,\hbox {d}^{-1}$, and for several values of the reproduction number $\rho$

Finally, note that the exact solution $i (\tau )$ of the SIR model is asymmetric. It rises very fast at first—exponentially actually—and then falls more slowly. The most explosive, severe outbreaks occur for $\rho \ge 5$, where the end of the epidemic is reached for $\tau _f\simeq 1$ (see Eq. (16)); thus, most of the initial susceptible individuals have been infected. For lower reproduction number the epidemic curve becomes more symmetrical and it is less explosive. Note that in the first wave of the COVID-19 pandemic daily deaths rose very rapidly and fell more slowly, indicating a high reproduction number, so that the data could not be fitted with logistic-type functions, which are symmetric, and a linear combination of two logistic functions was required to fit the data [18, 19, 28]. In the next section we will see that some more appropriate functions to describe this situation are the Gumbel functions, because they have the adequate asymmetry to fit almost correctly the solutions of SIR equations.

## Approximation to the SIR solutions with Gumbel functions

In Ref. [41] a Gumbel distribution was used to forecast the series of daily deaths with COVID-19 positives. The Gumbel distribution describes the probability of maximum (or minimum) values from the data of many observations [46–48]. It is not theoretically clear why the distribution of deaths or infections roughly recreates the distribution of maxima. In this section we will compare the Gumbel distribution to the exact solutions of the epidemiological SIR model. Both functions of time presents similar asymmetry and their parameters can be related numerically.

Although a Gumbel distribution does not exactly verify the SIR equation, the exact solution can be approximated quite well by the Gumbel distribution by choosing the parameters appropriately. In this section we will provide formulas that relate the parameters of SIR model with the parameters of an appropriate Gumbel function. For this end we will use the proper time method introduced in the previous section.

The Gumbel function is defined here as a time-dependent function with three parameters

26 $$\begin{aligned} G(t)= a \text {e}^{ -\text {e}^{-(t-t_0)/b} } \end{aligned}$$

and the derivative gives the Gumbel distribution

27 $$\begin{aligned} g(t)= \frac{\mathrm{d}{} \textit{G}}{\mathrm{d}{} \textit{t}}= \frac{1}{b}{\text {e}}^{-(t-t_0)/b}G(t). \end{aligned}$$

First, we will see how well the Gumbel distribution approximates the infected function of the SIR model, $g(t)\simeq i(t)$.

In Fig. 4 we compare the Gumbel distribution *g*(*t*) with the exact SIR solution *i*(*t*). The parameters of the Gumbel distribution have been fitted with least-squares method to obtain the optimal distribution that best describes the exact solution. The parameters of the SIR model are $s_{_0} = 0.99$, $\beta = 0.3$, and we use four values of $\rho = 1.5, 2, 3$ and 5—the same as in Fig. 3. The fitted parameters of the Gumbel distribution are given in Table 1.

**Table 1:** Parameters of the Gumbel distribution obtained by least squares fit to the exact solution of the SIR model, for four different values of the basic reproduction number $\rho$ (see Fig. 4)

$s_{_0}$	$\rho$	$\beta$	*a*	$t_0$	*b*
0.99	1.5	0.3	2.04	20.5	10.9
	2		2.67	14.9	6.29
	3		3.06	8.81	3.75
	5		3.12	5.30	2.44

The fits of Fig. 4 provide fairly good approximations to the exact SIR solutions with the Gumbel distribution. The results in Fig. 4 provide a validation of the Gumbel distribution to describe the evolution of an epidemic. Later, in Sect. 5 of results, we will see that this assessment is corroborated in the case of the description of the COVID-19 data. Although the fit is not perfect, the Gumbel distribution clearly shows similar asymmetry as the SIR model. The similarity is greater when the epidemic is explosive, with a high reproduction number $\rho \sim 3$–5. Where Gumbel fails most in these fits is in the initial and final stages of the Pandemic.

To investigate the connection between the Gumbel distribution and the solution of the SIR equations, it is convenient to express them as functions of proper time. First, we substitute *i*(*t*) by *g*(*t*) in the definition of proper time

28 $$\begin{aligned} \mathrm{d}\tau = \beta i \mathrm{d}t = \beta g \mathrm{d}t = \beta \frac{\mathrm{d}{} \textit{G}}{\mathrm{d}{} \textit{t}}\mathrm{d}t \end{aligned}$$

Thus integrating between 0 and *t* we have

29 $$\begin{aligned} \tau = \int _0^t \beta \frac{\mathrm{d}{} \textit{G}}{\mathrm{d}{} \textit{t}}\mathrm{d}t=\beta [G(t)-G_0] \end{aligned}$$

where $G_0=G(0)$. Hence

30 $$\begin{aligned} G(t)= G_0+\frac{\tau }{\beta }= a \text {e}^{ -\text {e}^{-(t-t_0)/b}} \end{aligned}$$

Taking the logarithm on both sides, we obtain

31 $$\begin{aligned} \text {e}^{-(t-t_0)/b} = \ln \frac{a}{G_0+\tau /\beta } \end{aligned}$$

from where we obtain the following approximate relation between *t* and $\tau$

32 $$\begin{aligned} t=t_0-b \ln \left( \ln \frac{a}{G_0+\tau /\beta }\right) , \end{aligned}$$

and we can write the Gumbel distribution as a function of $\tau$

33 $$\begin{aligned} g=\frac{1}{b} \text {e}^{-(t-t_0)/b}G = \frac{1}{b}\left( G_0+\frac{\tau }{\beta }\right) \ln \frac{a}{G_0+\tau /\beta }\nonumber \\ \end{aligned}$$

Starting with this expression as a function of proper time, we can consider several alternative ways of adjusting the Gumbel parameters from the parameters of the SIR model, assuming that the proper time is the same in both models.

### Proper time fit 1

The idea of the proper time fit consists in imposing that the maximum of $g(\tau )$ coincides with the maximum of $i(\tau )$. In fit 1, we will also assume that $G_0$ is very small can be neglected, $G_0 \simeq 0$, i.e., we will not try to impose any additional condition on the initial value. This allow us to estimate the parameters *a* and *b* easily, but we will not be able to obtain the value of $t_0$, which will later be adjusted to fit the temporal peak of the SIR solution.

We start by writing Eq. (33) for $G_0=0$

34 $$\begin{aligned} g(\tau )= \frac{\tau }{b \beta }\ln \frac{a\beta }{\tau }. \end{aligned}$$

To find the maximum of this function, we compute the derivative

35 $$\begin{aligned} g'(\tau )= \frac{1}{b \beta }\ln \frac{a\beta }{\tau }-\frac{1}{b \beta } \end{aligned}$$

from where we find the maximum condition

36 $$\begin{aligned} \ln \frac{a\beta }{\tau }=1. \end{aligned}$$

Thus, the value of the peak position, $\tau _\text {peak}$, is

37 $$\begin{aligned} \tau _\text {peak}= \frac{a\beta }{\text {e}} \end{aligned}$$

and the height of the peak (maximum of *g*) is

38 $$\begin{aligned} g( \tau _\text {peak})= \frac{1}{\text {e}}\frac{a}{b} \end{aligned}$$

Comparing to the peak values of the SIR solution, Eqs. (19, 21), and equating the values we obtain

39 $$\begin{aligned} \frac{a\beta }{\text {e}}= & {} \frac{\ln s_{0}\rho }{\rho } \end{aligned}$$

40 $$\begin{aligned} \frac{1}{\text {e}}\frac{a}{b}= & {} 1- \frac{1+\ln s_{0}\rho }{\rho }. \end{aligned}$$

From here we obtain the values of the parameters *a* and *b* of the Gumbel distribution in terms of the parameters of the SIR model

41 $$\begin{aligned} a= & {} \frac{{\text {e}} \ln s_{_0}\rho }{\lambda } \end{aligned}$$

42 $$\begin{aligned} \frac{a}{b}= & {} \text {e} \left( 1- \frac{1+\ln s_{_0}\rho }{\rho } \right) \end{aligned}$$

We see that the values of *a* and *b* can be calculated directly from the parameters and initial conditions of the SIR model. The value of $t_0$ would be fitted later to the position of the peak as a function of time.

Note that if the initial number of infected individual is very small, then $s_{_0}$ can be approximated by one in the logarithm $\ln s_{_0}\rho \simeq \ln \rho$. Then the parameters *a* and *b* do not depend appreciably on the precise value of $s_{_0}$, but only on $\rho$ and $\lambda$ by the simple relations

43 $$\begin{aligned} a= & {} \frac{\text {e} \ln \rho }{\lambda } \end{aligned}$$

44 $$\begin{aligned} \frac{a}{b}= & {} \text {e} \left( 1- \frac{1+\ln \rho }{\rho } \right) \end{aligned}$$

Note also that Eq. (44) can be solved numerically to obtain the reproduction number $\rho$ as a function of *a*/*b*, and then Eq. (43) gives $\lambda$. So, the constants $\lambda$ and $\rho$ of the SIR system can also be computed from the values of the constants *a* and *b* of the Gumbel distribution.

**Fig. 5:**
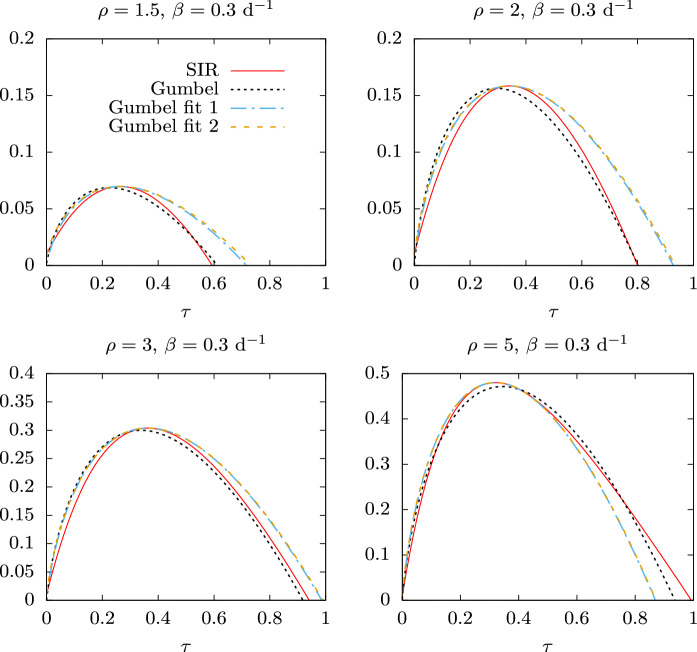
Three Gumbel distributions as a function of the proper time, compared to the exact Solution of the SIR equations for $s_{_0}=0.99$, $\beta =0.3\,\hbox {d}^{-1}$, and for several values of the reproduction number $\rho$. The parameters of the Gumbel distributions correspond to the mean-square fit, proper-time fit 1 and proper-time fit 2. In proper-time fit 1, the value of $t_0$ is taken from Table 1

**Fig. 6:**
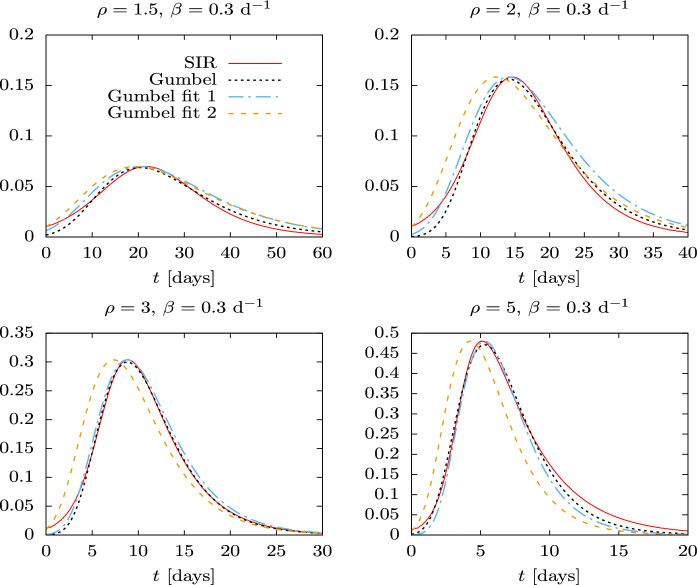
Three Gumbel distributions compared to the exact Solution of the SIR equations for $s_{_0}=0.99$, $\beta =0.3\,\hbox {d}^{-1}$, and for several values of the basic reproduction number $\rho$. The parameters of the Gumbel distributions correspond to the mean-square fit, proper-time fit 1 and proper-time fit 2. In proper-time fit 1, the value of $t_0$ is taken from Table 1

### Proper time fit 2

A second fit can be done by fixing also the number of initial infected *i*(0), in which case a value for $t_0$ can be theoretically obtained. We proceed as in previous section, by computing the maximum of the Gumbel distribution, as a function of proper time, but in this case we use the exact expression, Eq. (33). The maximum of $g(\tau )$ is now obtained for

45 $$\begin{aligned} \ln \frac{a}{G_0+\tau /\beta }=1 \end{aligned}$$

from where the peak (maximum of *g*) is reached at the proper time

46 $$\begin{aligned} \tau _\text {peak}= \frac{a\beta }{\text {e}}G_0\beta \end{aligned}$$

and the value of the maximum is

47 $$\begin{aligned} g(\tau _\text {peak})= \frac{1}{\text {e}} \frac{a}{b} \end{aligned}$$

this is the same value for the peak obtained in Eq. (38).

Comparing to the peak values of the SIR solution, Eqs. (19, 21), and equating the values we obtain

48 $$\begin{aligned}&\frac{a\beta }{\text {e}} -G_0\beta = \frac{\ln s_{_0}\rho }{\rho } \end{aligned}$$

49 $$\begin{aligned}&\frac{1}{\text {e}}\frac{a}{b} = 1- \frac{1+\ln s_{_0}\rho }{\rho } \end{aligned}$$

Equation (49) gives the same value for *a*/*b* obtained in fit 1. For $s_{_0}\simeq 1$, this value only depends on the basic reproduction number $\rho$.

A third condition is obtained if we demand $g(0)=i(0)=1-s_{_0}$. Using Eq. (33) for the Gumbel distribution as a function of proper time, we have

50 $$\begin{aligned} g(0)=\frac{1}{b}\ln \frac{a}{G_0}G_0=1-s_{_0} \end{aligned}$$

where

51 $$\begin{aligned} G_0= a\text {e}^{-\text {e}^{t_0/b}}=a\text {e}^{-x} \end{aligned}$$

where we have defined the parameter

52 $$\begin{aligned} x=\text {e}^{t_0/b}. \end{aligned}$$

In terms of the *x*-parameter, the initial value condition can be written as

53 $$\begin{aligned} g(0)=\frac{a}{b}x\text {e}^{-x}. \end{aligned}$$

The procedure of fit 2 follows the following steps:

1. Compute *a*/*b* from Eq. (49)
  54 $$\begin{aligned} \frac{a}{b} = \text {e}\left( 1- \frac{1+\ln s_{_0}\rho }{\rho }\right) . \end{aligned}$$
2. Write the initial condition $g(0)=i(0)$ as
  55 $$\begin{aligned} x\text {e}^{-x}=\frac{b}{a}( 1-s_{_0}) \equiv \epsilon _0 \end{aligned}$$
  and solve this equation numerically for $x>0$
3. Once we know the values of *x* and *a*/*b*. compute the value of *a* using Eq. (48)
  56 $$\begin{aligned} a= \frac{\ln s_{_0}\rho }{\lambda (\text {e}^{-1}-\text {e}^{-x})} \end{aligned}$$
4. Compute $b=(b/a) a$, and finally
5. Compute $t_0$ as
  57 $$\begin{aligned} t_0= b\ln x. \end{aligned}$$

In Figs. 5 and 6 the solution of the SIR model is compared with the Gumbel distributions corresponding to the three fits considered in this work: the least square fit, and the proper time fits 1 and 2.

The least square fit, with the parameters of Table 1, is represented with dotted lines as a function of proper time in Fig. 5 and is very close to $i(\tau )$. We see in Fig. 5 that the proper time fit 1 and fit 2 are essentially the same and that their maximum coincides (by construction) with the maximum of the SIR solution $i(\tau )$. The parameter $t_0$ in the case of fit 1 is taken from Table 1, because it cannot be obtained theoretically. The parameter $t_0$ in fit 2 is computed from Eq. (57) in terms of the computed values of *b* and *x*.

We see that fit 1 and 2 in general do not give the end time $\tau _f$ of the epidemic correctly. This is because in these fits only the position of the maximum and the maximum value of $i(\tau )$ are adjusted, in addition to the initial value *i*(0) in fit 2, but they have not been adjusted to give the final point $\tau _f$ of the epidemic.

For low values of the basic reproduction number, $\rho =1.5{-}3$, both fit 1 and fit 2 are wider than $i (\tau )$ in Fig. 5, and they extend at the end of the epidemic up to a proper time larger than the exact solution. When $\rho$ is larger the width of the Gumbel functions of fits 1 and 2 begin to decrease in relation to the SIR result, until their widths become smaller than the width of the SIR solution, for $\rho = 5$.

The results of Fig. 5 are translated into the distributions as a function of physical time in Fig. 6. The Gumbel fits 1 and 2 differ mainly in the value of $t_0$. The maximum of fit 2 is shifted to the left with respect to the maximum of fit 1. This happens because in fit 2 we are demand that the initial value of the Gumbel distribution be equal to *i*(0). As a result *g*(*t*) is shifted to the left of *i*(*t*), because the Gumbel distribution increases slightly faster than the SIR solution as a function of time. By construction, The maximum number of infected coincides in fits 1 and 2 with the maximum of *i*(*t*), but it occurs slightly earlier in fit 2.

With appropriate parameters we have seen that the Gumbel distribution approximately describes the exact solution of the SIR model. Although the description is not perfect, the Gumbel distribution has a width and asymmetry similar to those of the epidemiological curve *i*(*t*), which allows fitting data of an epidemic. Subsequently, once the Gumbel distribution has been fitted to data, the parameters of the SIR model can be obtained as a first approximation using the formulas in this section. The fact that the Gumbel distribution is analytical as a function of time allows its parameters to be easily fitted, unlike the SIR model, which, although simple, must be solved numerically.

**Fig. 7:**
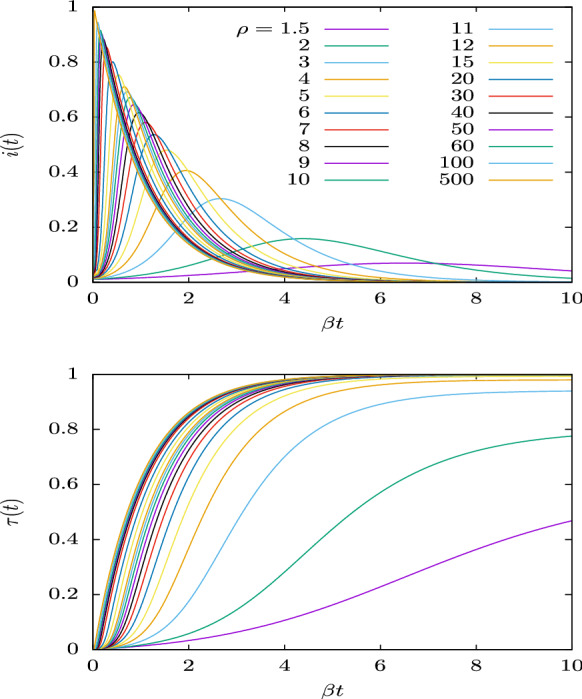
Solutions of the SIR equations for values of the basic reproduction number $\rho$ from 1.5 to 500. The functions *i*(*t*) and $\tau (t)=r(t)$ are Plotted as a function of the normalized time $\beta t$

**Fig. 8:**
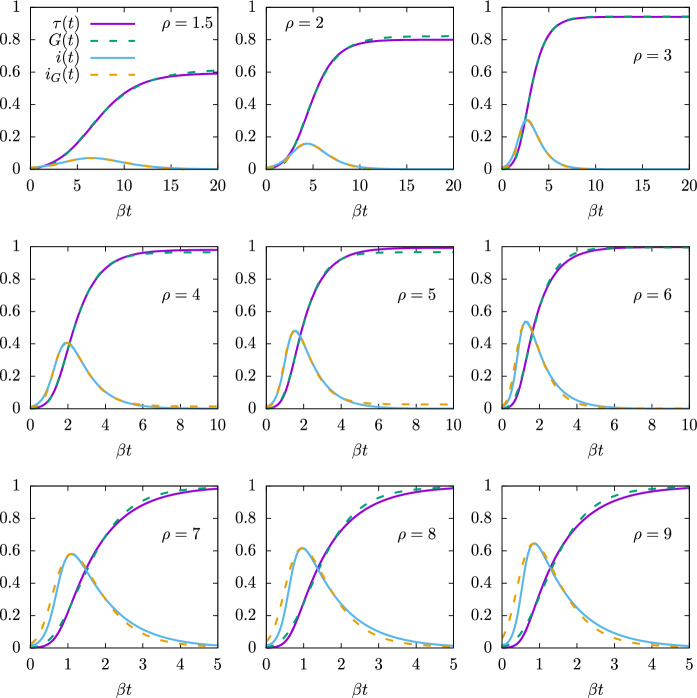
Solutions of the SIR equations for several values of the basic reproduction number $\rho$, and for $s_{_0}=0.99$. The functions *i*(*t*) and $\tau (t)=r(t)$ are plotted as a function of the normalized time $\beta t$. These are compared to the approximate solution obtained with a Gumbel function *G*(*t*) fitted to $\tau (t)$, and with the corresponding infected function $i_G(t)= 1- G(t)-s_{_0}\exp (-\rho G(t))$ of the extended SIR model

## Asymmetry of the SIR solutions

An essential characteristic of the solution *i*(*t*) of the SIR equations is the evident asymmetry that this function presents with respect to its maximum value. The asymmetry is more pronounced as the reproduction number $\rho$ increases. Indeed, in this section we will define a parameter that uniquely characterizes the asymmetry and we will see that said asymmetry increases linearly with the basic reproduction number $\rho$

We begin by mentioning an important property of the *i*(*t*) solution concerning the scaling property with respect to time. It is clearly seen in Eq. (25) that the physical time as a function of proper time, $t(\tau )$, is inversely proportional to $\beta$. Therefore any change in $\beta$ translates into a change in the time scale. The natural scale of time is thus $\beta t$, that is, measuring time in units of $1/\beta$. Thus, from the solutions of the SIR equations for $\beta =1$, all the others are obtained simply by re-scaling the time with a factor $1/\beta$.

Therefore in Fig. 7, where we represent the solutions of the SIR equations for a range of $\rho$ values from 1.5 to 500, and for $\beta =1$, all the solutions are included if they are represented as a function of $\beta t$. Note that here we use the initial condition $s_{_0} = 0.99$. But this does not detract from the generality of our affirmations, because a change in the initial condition ultimately translates into a shift of the time origin such that $i(0)=0.01$.

Figure 7 shows that as $\rho$ increases, the epidemic progresses faster, that is, it ends earlier. As a function of natural time (or for $\beta =1$ day$^{-1}$), it lasts from about 20 days for $\rho =1.5$, to less than a week for $\rho >10$, and only a few days if $\rho > 20$. Moreover, for very large values of $\rho >10$, the epidemic grows very quickly in a short time interval and then decreases exponentially-like regardless of the value of $\rho$, with $i(t) \simeq e^{-\beta t}$. In Fig. 7 (top panel) it is also evident that the asymmetry of *i*(*t*) increases with $\rho$.

The recovered function, or proper time, $\tau (t)=r(t)$, is displayed in the bottom panel of Fig. 7. For small values of $\rho$, it quite resembles a Gumbel function, but for very large values of the basic reproduction number, $\rho > 10$, it departs from the family of Gumbel functions and approaches the function $r(t)= 1-\exp (-\beta t)$, as the limit for $\rho \rightarrow \infty$.

The behavior of the SIR solution and the Gumbel functions can be seen more clearly in Fig. 8. There we plot the infected and recovered functions as a function of $\beta t$, for the SIR model and fitting a Gumbel function, for $\rho =1.5,2,3 \ldots ,9$. In Fig. 8, the fit is an alternative to the one made in Sect. 2. There, the Gumbel distribution *g*(*t*) was fitted directly to the infected function *i*(*t*). In Fig. 8 we first fit the Gumbel function *G*(*t*) to the recovered function $\tau (t)=r(t)$ by least squares method, and then we calculate the Gumbel-infected function using the SIR Eq. (15)

58 $$\begin{aligned} i_G(t) = 1- G(t) - s_o \exp (-G(t )) \end{aligned}$$

for $\beta =1$. The results of fitting *G*(*t*) are not exactly the same as fitting *g*(*t*), because a different function if being fitted in each case, but they are very similar.

We notice that for $\rho =5$, the resulting function $i_G(t)$ does not converges to zero for large *t*. This happens because the least-squares fit of the Gumbel function does not exactly approach the maximum value of $\tau (t)$. To remedy this, starting $\rho =6$, we set the value of the *a* parameter of *G*(*t*) so that $G(t)=\tau (t)$ for large *t*, and then fit the two parameters *b* and $t_0$.

Figure 8 shows that the Gumbel distribution is very similar to the SIR solution, but starting from $\rho =7$, small differences begin to be seen because the asymmetry of Gumbel and the SIR solution begins to diverge from each other. For $\rho =9$ such a difference is already quite appreciable, and it increases for larger values of $\rho$.

**Fig. 9:**
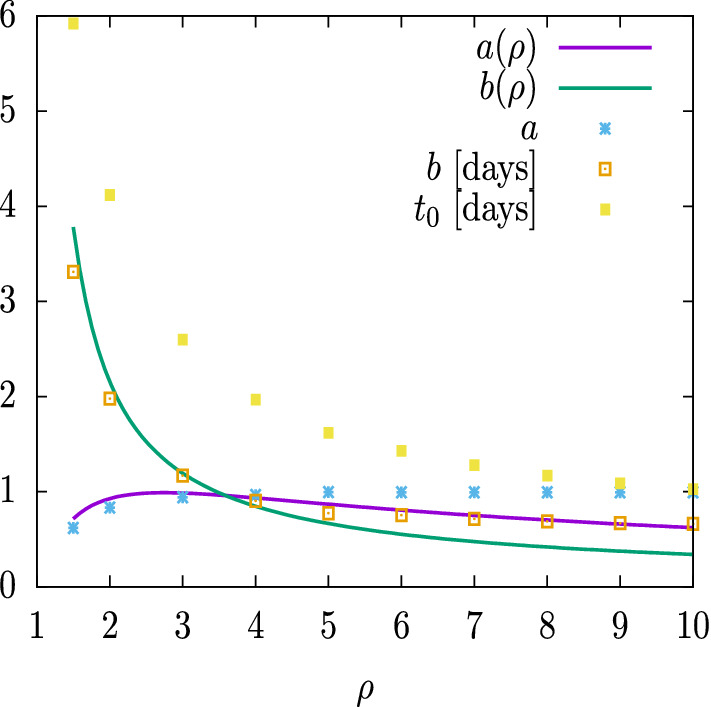
Parameters of the Gumbel function *G*(*t*) fitted to $\tau (t)$ in Fig. 8. These are compared to the functions $a(\rho )$ and $b(\rho )$ from Eqs. (39, 40)

In Fig. 9 we show the values of the Gumbel parameters, $a, b, t_0$, fitted in Fig. 8, as a function of the reproduction number $\rho$. We also show the values of $a(\rho )$ and $b(\rho )$ calculated with Eqs. (39) and (40), which give an analytical approximation between the Gumbel function and the SIR solution. We see that the values of a and b are very close to their analytic approximations for small $\rho$, and that they start to differ from $\rho \sim 5{-}6$ this indicates that the approximation of Eqs. (39, 40) worsens for very high values of $\rho$. This difference is due in part to the fact that in Eqs. (39) and (40) they were obtained by requiring that the maxima of *i*(*t*) and *g*(*t*) coincide. But in Fig. 8 we have adjusted the parameter *a* so that the maximum of *r*(*t*) coincides with the maximum of *G*(*t*) and that alters Eqs. (39) and (40).

Next we propose a definition of the asymmetry of the SIR solution in terms of the half-widths of the distribution *i*(*t*) to the left and to the right of the maximum $i_{\mathrm{peak}}=i(\tau _{\mathrm{peak}})$. First we define $\tau _1$ and $\tau _2$ as the values of the proper time such that $i(\tau _1)= i(\tau _2)= i_{\mathrm{peak}}/2$ and $\tau _1<\tau _{\mathrm{peak}}<\tau _2$. Specifically, $\tau _1$ and $\tau _2$ are the two solutions of the equation

59 $$\begin{aligned} 1-\tau -s_{_0}\text {e}^{-\rho \tau }= \frac{i_{\mathrm{peak}}}{2} \end{aligned}$$

where $\tau _{\mathrm{peak}}$ and $i_{\mathrm{peak}}$ corresponds to the position and value of the maximum of $i(\tau )$, given by Eqs. (19, 20). Therefore, $\tau _1$ and $\tau _2$ are the proper-time values at which $i(\tau )$ reaches half of its maximum value. Two half-widths are defined in term of the physical time as

60 $$\begin{aligned} \Gamma _1= & {} \beta (t(\tau _{\mathrm{peak}})-t(\tau _1))\end{aligned}$$

61 $$\begin{aligned} \Gamma _2= & {} \beta (t(\tau _2) - t(\tau _{\mathrm{peak}})) \end{aligned}$$

In view of the previous numerical results, we see that $\Gamma _2 > \Gamma _1$ for $\rho >1$, that is, the SIR solution is wider to the right than to the left of the maximum. The asymmetry is then defined as the quotient

62 $$\begin{aligned} {{\mathcal {A}}}(\rho )= \frac{\Gamma _2}{\Gamma _1} \ge 1. \end{aligned}$$

This function increases with $\rho$. This asymmetry is defined as a ratio that does not depend on $\beta$ nor on the global normalization of *i*(*t*). Therefore, it is a suitable parameter to express the asymmetry of the theoretical distribution, as well as that of the experimental data of deaths described in the next section.

**Fig. 10:**
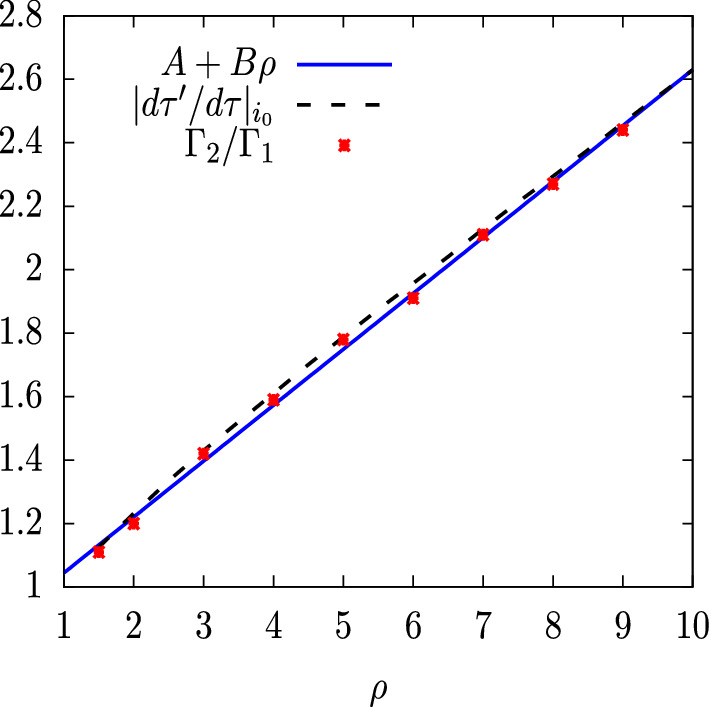
Asymmetry of the SIR solutions as a function of $\rho$. The points are the values of the asymmetry computed numerically for discrete values of $\rho =1.5, 2, 3,\ldots ,9$. The solid line is a fitted right line $A+B\rho$. The dashed line is the Jacobian $|\mathrm{d}\tau '/\mathrm{d}\tau |_{i_{_0}}$ evaluated for $i_{_0}=0.84 i_{\mathrm{peak}}$

To compute the asymmetry of the SIR solution, for a $\rho$ value, we fist solve the transcendental equation (59) numerically, and obtain the two solutions $\tau _1 < \tau _2$. The half widths are then computed as the integrals

63 $$\begin{aligned} \Gamma _1= & {} \int _{\tau _1}^{\tau _{\mathrm{peak}}}\frac{\mathrm{d}\tau }{1-\tau -s_{_0}\text {e}^{-\rho \tau }} \end{aligned}$$

64 $$\begin{aligned} \Gamma _2= & {} \int _{\tau _{\mathrm{peak}}}^{\tau _2}\frac{\mathrm{d}\tau }{1-\tau -s_{_0}\text {e}^{-\rho \tau }} \end{aligned}$$

These integrals are computed numerically and the corresponding asymmetry is plotted in Fig. 10 for $\rho =1.5,2,3,\ldots ,9$, and for $s_o=0.99$. We see that, as a function of $\rho$, the asymmetry of the SIR solutions is quite approximately a linear function of $\rho$, which is very well fitted by the parametrization ${{\mathcal {A}}}= A + B\rho$, with $A=0.868$ and $B=0.176$, as seen in Fig. 10.

To better understand why this quasi-linear dependence on asymmetry occurs, we proceed as follows. A rigorous proof is not possible to our understanding because transcendental equations are involved, and because the linearity is only approximate, but its origin can be roughly understood.

First, in the interval $[\tau _1,\tau _{\mathrm{peak}}]$ the function $i(\tau )=1-\tau -s_{_0}\text {e}^{-\rho \tau }$ is increasing and the change of variable $\tau \rightarrow i$ can be made inside the integral $\Gamma _1$, with Jacobian $\mathrm{d}i/\mathrm{d}\tau = \rho s_{_0}\text {e}^{-\rho \tau }-1 >0$. We obtain

65 $$\begin{aligned} \Gamma _1 = \int _{i_{\mathrm{peak}}/2}^{i_{\mathrm{peak}}}\frac{\mathrm{d}\tau }{di} \frac{\mathrm{d}i}{i} \end{aligned}$$

Second, in the interval $[\tau _{\mathrm{peak}},\tau _2]$ the function $i(\tau ')=1-\tau '-s_{_0}\text {e}^{-\rho \tau '}$ is decreasing and the change of variable $i\rightarrow \tau '$ can be made inside the integral (65), with Jacobian $\mathrm{d}i/\mathrm{d}\tau '= \rho s_{_0}\text {e}^{-\rho \tau '}-1 <0$. We obtain

66 $$\begin{aligned} \Gamma _1=\int _{\tau _2}^{\tau _{\mathrm{peak}}}\frac{\mathrm{d}\tau }{di}\frac{\mathrm{d}i}{\mathrm{d}\tau '} \frac{\mathrm{d}\tau '}{i(\tau ')}= \int _{\tau _2}^{\tau _{\mathrm{peak}}}\frac{\mathrm{d}\tau }{\mathrm{d}\tau '} \frac{\mathrm{d}\tau '}{i(\tau ')} \end{aligned}$$

with

67 $$\begin{aligned} \frac{\mathrm{d}\tau }{\mathrm{d}\tau '}= \frac{\mathrm{d}\tau }{di} \frac{\mathrm{d}i}{\mathrm{d}\tau '}= \frac{ \rho s_{_0}\text {e}^{-\rho \tau '}-1 }{\rho s_{_0}\text {e}^{-\rho \tau }-1}<0\, . \end{aligned}$$

This Jacobian corresponds to the change of variable $\tau \rightarrow \tau '$, inside $\Gamma _1$, where $\tau <\tau '$ are the two solutions of the transcendental equation $i=1-\tau -s_{_0}\text {e}^{-\rho \tau }$, for a fixed value of $i \in [i_{\mathrm{peak}}/2,i_{\mathrm{peak}}]$. Note that this is the interval of integration in Eq. (65) where we are changing the variable $i \rightarrow \tau '$.

Now, using the mean value theorem, we can factor the Jacobian out of the second integral of Eq. (66), evaluated at some intermediate value of $i=i_{_0}$ between $i_{\mathrm{peak}/2}$ and $i_{\mathrm{peak}}$, obtaining

68 $$\begin{aligned} \Gamma _1 =\left| \frac{\mathrm{d}\tau }{\mathrm{d}\tau '}\right| _{i_{_0}} \int _{\tau _{\mathrm{peak}}}^{\tau _2} \frac{\mathrm{d}\tau '}{i(\tau ')} =\left| \frac{\mathrm{d}\tau }{\mathrm{d}\tau '}\right| _{i_{_0}} \Gamma _2 \end{aligned}$$

We have numerically calculated the Jacobian $|\mathrm{d}\tau '/\mathrm{d}\tau |$ for different values of $i_0$ and we have compared it with the asymmetry $\Gamma _2/\Gamma _1$. Both coincide approximately for $i_0=0.84 i_{\mathrm{peak}}$ regardless of the value of $\rho$. This can be seen in Fig. 10 where we represent the Jacobian as a function of $\rho$, for $i_0=0.84 i_{\mathrm{peak}}$. We see that quite a straight line is obtained that coincides with the calculated value of the asymmetry.

The quasi-linear behavior of the asymmetry of the SIR solutions as a function of $\rho$ allows us to obtain the value of $\rho$ from experimental data, after estimating the asymmetry empirically.

**Fig. 11:**
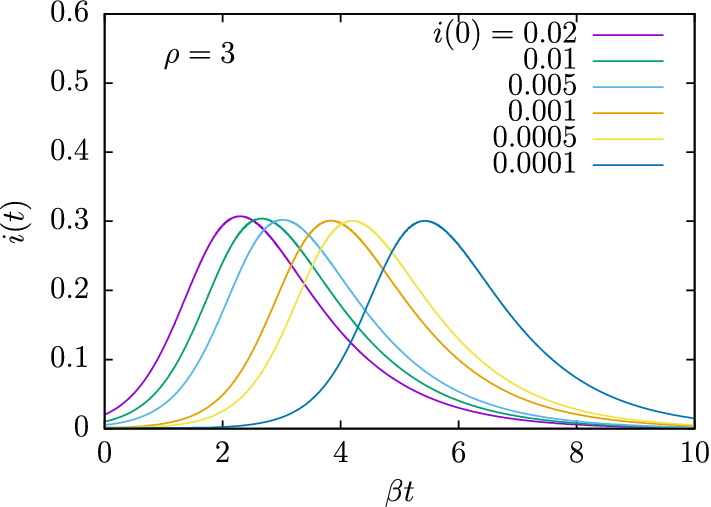
The function *i*(*t*) obtained for $\rho =3$, and computed for different initial values of *i*(0)

To finish, note that in the theoretical development of this and the previous sections we have assumed that $r(0)=0$ for $t=0$, and for this reason the initial value of $\tau$ is zero also for $t=0$. This technique is then applicable for the early stages of the epidemic when the number of recoveries is not yet significant. It is also expected that the initial value of those infected is not very high in the first days of the epidemic, and in the previous mathematical developments we have studied the particular case $i(0)=1-s_0=0.01$, that is, 1% of the population is initially infected. For other initial values of *i*(0) the results are equally valid, as long as the condition $r(0)=0$ holds. The effect of a change in the value of *i*(0) is to produce a shift with respect to time of the SIR model solutions. This can be verified in the results of Fig. 11. There we show the solutions of the function *i*(*t*) for $\rho =3$ and for different values of the initial value of infected $i(0)=0.02$, 0.01, 0.005, 0.001, 0.0005, 0.0001. Indeed we see that by changing the initial condition *i*(0) the function *i*(*t*) is basically the same shifted in time. Therefore, the theoretical results about the properties of the SIR model solutions, and in particular the asymmetry properties of *i*(*t*), do not depend on *i*(0).

**Table 2:** Parameters of the Gumbel and SIR models fitted for the different countries considered in this work

Country	*G*(*t*)	*g*(*t*)	SIR			*g*(*t*) fit to SIR
	*b* [d]	*b* [d]	$\rho$	$\beta$ [d$^{1}$]	$\beta ^{-1}$ [d]	*b* [d]
Spain	13.4	12.6	7	0.05	20	12
UK	17.7	16.15	7	0.038	26	16.2
France	13.35	11.6	6	0.06	16.7	10.8
Italy	18.4	17.1	5.5	0.045	22.2	15.2
Germany	15.4	14.6	5.5	0.05	20	13.6
Canada	18.01	18.1	5.1	0.041	24.4	16.9
Belgium	12.8	11.65	3	0.1	10	11.27
Switzerland	11.7	11.79	4	0.085	11.8	10.16
Sweden	26.7	25.66	7	0.03	33	18.8
USA	21.1 [0:150]	19.6	8	0.028	35.7	17.7
		47.4 (2nd wave)				
Brazil	60.6 [0:250]	35.3				
		37 (2nd wave)				
India	60	66.4				
		32 (2nd wave)				

**Fig. 12:**
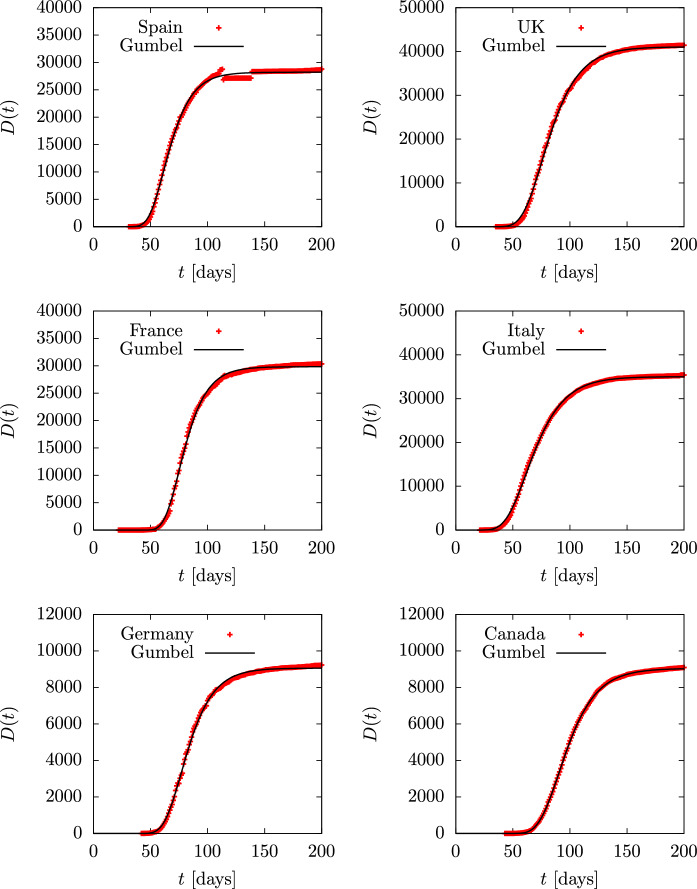
Total deaths *D*(*t*) during the first wave of the COVID-19 pandemic for several countries, compared to the Gumbel function *G*(*t*). The parameter *b* of the Gumbel function fitted to the data is given in Table 2. Data are from Ref. [49]

**Fig. 13:**
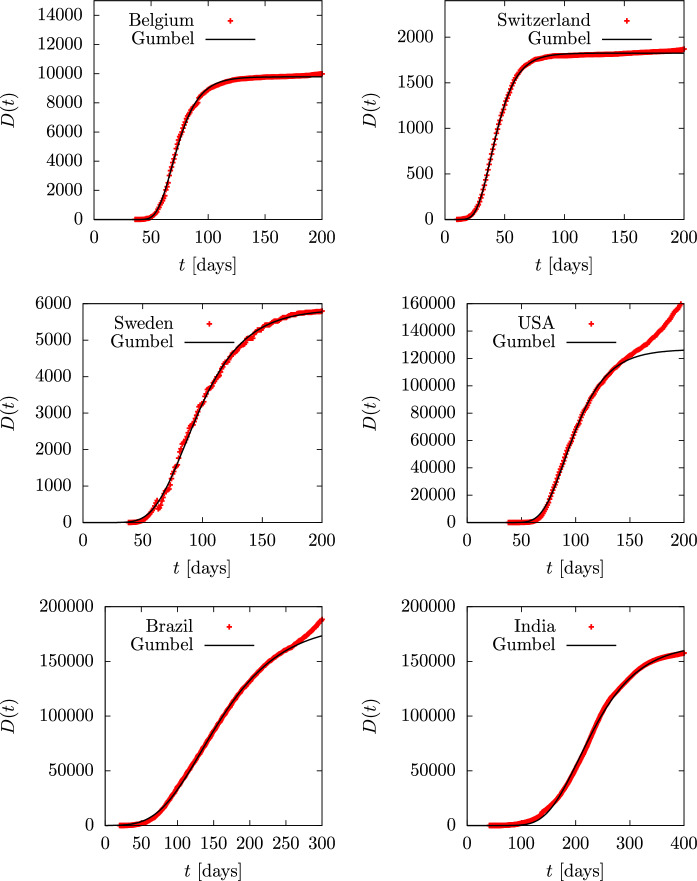
Total deaths *D*(*t*) during the first wave of the COVID-19 pandemic for several countries, compared to the Gumbel function *G*(*t*). The parameter *b* of the Gumbel function fitted to the data is given in Table 2. Data are from Ref. [49]

## Results

In this section we present results comparing with the data of total deaths *D*(*t*) and daily deaths $\Delta D (t)$ as a function of time for the first wave of the COVID-19 pandemic. These data are supplied by government ministries in different countries and are available from various sources. The data can vary in different sources, as it can often be found averaged or smoothed, or in many cases the data can be later revised and updated differently at different sites.

To study the daily deaths, we are assuming that a certain constant portion of removals ends in death, that is, the death rate verifies an equation similar to the removal rate with a different constant

69 $$\begin{aligned} \frac{\mathrm{d}{} \textit{G}}{\mathrm{d}{} \textit{t}} = \alpha I. \end{aligned}$$

this means that the daily deaths, $\Delta D(t) \simeq \frac{\mathrm{d}D}{\mathrm{d}}$, are described, except normalization constant, by the solution *i*(*t*) of the SIR equations and we can use the formalism of the previous sections.

This study could also be carried out including infected cases, but we have chosen to study deaths because the real number of infected is not known, since only detected cases are reported. On the other hand, the number of deaths also has that uncertainty. In any case, we work with the hypothesis that the number of real deaths is statistically proportional to that reported and that proportionality is somehow included in the $\alpha$ constant of Eq. (69).

One of our purposes of this work is to check if the SIR model is capable of describing the data, in which case it can be assumed that the hypotheses of this very simple model are justified; in particular if averaged values can be adopted for the two basic constants of the model: the reproduction number $\rho$ and the recovery rate $\beta$. This would indicate the universal validity of the SIR model. Comparing the parameters of the model between different countries will tell us the degree of universality of these parameters when passing from one country to another.

**Fig. 14:**
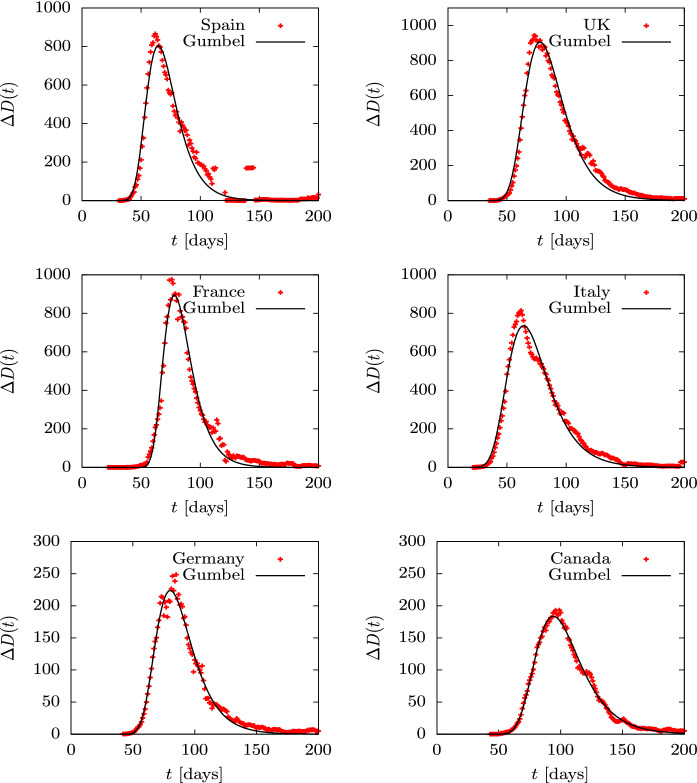
Daily deaths $\Delta D(t)$ during the first wave of the COVID-19 pandemic for several countries compared to the Gumbel function *G*(*t*). The parameter *b* of the Gumbel function fitted to the data is given in Table 2. Data are from Ref. [49]

**Fig. 15:**
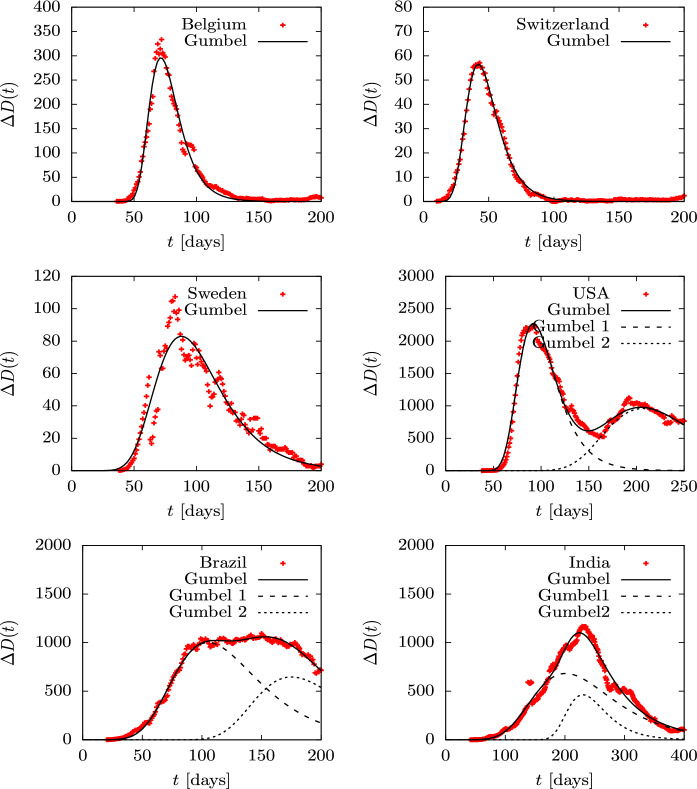
Daily deaths $\Delta D(t)$ during the first wave of the COVID-19 pandemic for several countries compared to the Gumbel function *G*(*t*). The parameter *b* of the Gumbel function fitted to the data is given in Table 2. Data are from Ref. [49]

**Fig. 16:**
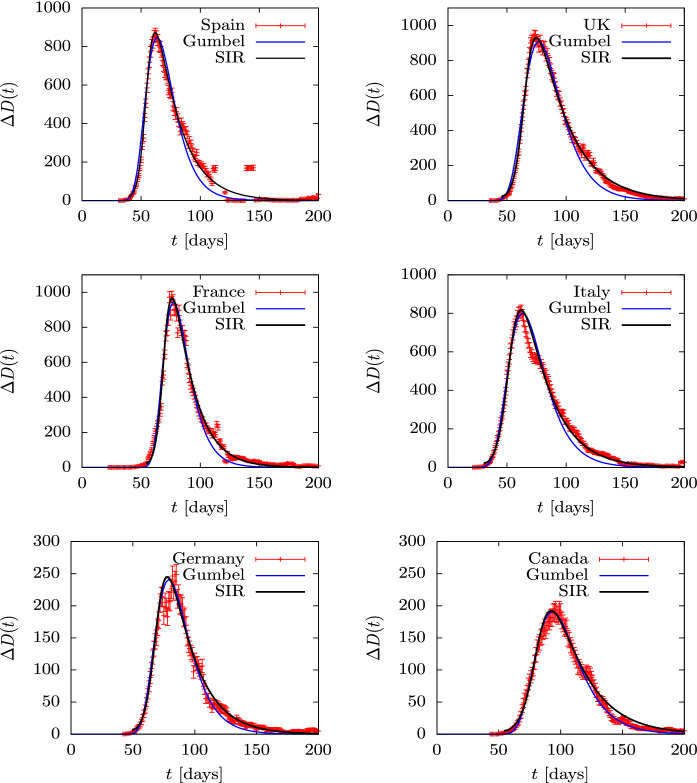
Daily deaths $\Delta D(t)$ during the first wave of the COVID-19 pandemic for several countries compared to the Gumbel function *G*(*t*). The parameter *b* of the Gumbel function fitted to the data is given in Table 2. Data are from Ref. [49]

**Fig. 17:**
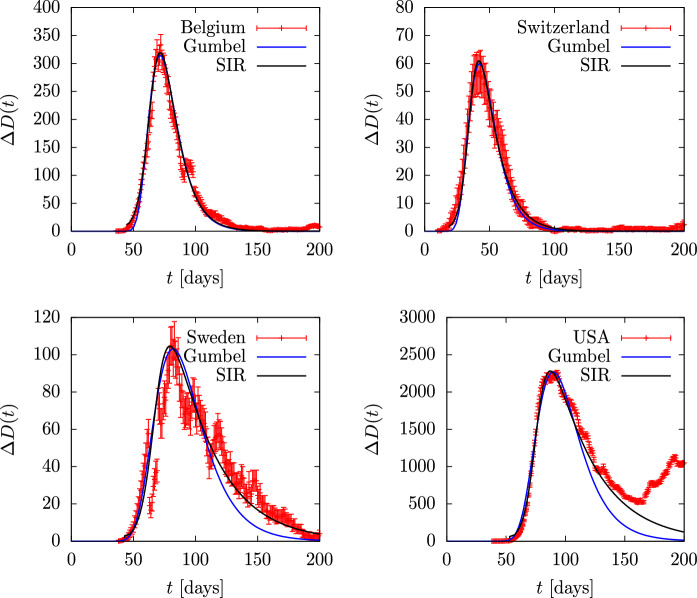
Daily deaths $\Delta D(t)$ in the first wave of coronavirus pandemic for several countries, compared to the Gumbel function *G*(*t*). The parameter *b* of the Gumbel function fitted to the data is given in Table 2. Data are from Ref. [49]

For the present study we have selected the countries where the first wave of the COVID-19 pandemic is visually similar to the solution of the SIR equations. The data that we have studied come from Ref. [49]. But they can also be found in other sources, such as the worldometer website [40]. We have opted not to use the WHO data [50] because often show very large daily fluctuations. The data from Ref. [49] are smoother, having been updated or averaged, and are more suitable for this study.

After inspecting the data for each country, we have selected ten countries where the series of daily deaths resemble the solutions of the SIR equations or equivalently to the Gumbel distribution, with a more or less asymmetric bell shape. The rest of the countries have a more irregular shape (probably due to a deficient counting system) or else there are not enough data to be able to be statistically described with the equations of the SIR model. The selected countries are: Spain, France, Italy, UK, Germany, Canada, Belgium, Switzerland, Sweden and USA (we left aside the curious case of China where the epidemic ended mysteriously and prematurely). Here we only consider the first wave of the pandemic, because the subsequent ones are much more irregular and require a separate study. Note that we only analyze the data on daily deaths and cumulative deaths. The homogeneity of the reported number of infected individuals is questioned because it is proportional to the number of tests performed, and the experimental error of the tests is not given.

To begin with, in Figs. 12 and 13 we show the accumulated deaths as a function of time in the ten countries mentioned with the addition of Brazil and India, as they are the top countries in number of infections and perhaps also in number of deaths. Time is measured in days. Data are from Ref. [49] and day one is 2020 February 1. A Gumbel function has been fitted for each country. The only tabulated parameter in the second column of Table 2 is the value of the *b* parameter in days. Note that the value of *a* in the Gumbel function is simply a normalization constant, and the value of $t_0$ is a shift in time. Thus what really characterizes the dynamics is the value of the parameter *b*, which is related to the duration of the epidemic. The USA data have been fitted until day 150, when the second wave starts to appear. In the case of Brazil and India the fit is performed until day 250. Note that Spain, France and Belgium and Switzerland have similar *b*-values in the range $b\sim 12{-}13$ days. Italy, UK and Canada are well fitted with $b\sim 18$ days. Germany is in between with $b\sim 15$, and Sweden is the European country with the highest value of $b\sim 27$ days.

Cumulative deaths, *D*(*t*), are fairly smooth distributions and very similar among different countries because we are dealing with sums —or integrals on the continuous limit. More detailed information is obtained by describing the daily death data $\Delta D(t)$, shown in Figs. 14 and 15. Although these data show more fluctuations, they can be fitted well with the Gumbel *g*(*t*) distribution, although the fit parameters differ somewhat from those obtained by fitting the Gumbel function *G*(*t*), since different functions and data are being involved. the fitted parameter *b* is in the third column of Table 2. Again we only tabulate the parameter *b*, because *a* and $t_0$ give simply the relative height and the position of the peaks. in the case of the USA, Brazil and India, we fit two Gumbel distributions, since it is apparent that there are at least two overlapping waves. In these cases, in Table 2 we tabulate the two values $b_1$ and $b_2$ of the two fitted waves.

We see that all countries are well fitted with one or two Gumbel distributions, so this function is an optimal candidate to quantitatively describe an epidemic of these characteristics with only one parameter, *b*, plus the normalization and position of the peak. The fact that the nine countries considered with an isolated first wave (Spain, France, Italy, UK, Germany, Canada, Belgium, Switzerland and Sweden) only require a time-independent parameter is remarkable. This does not happen in the following waves or in other countries, where the data show different behavior with large overlaps and stochastic fluctuations.

Since Gumbel provides a good analytical approximation to the SIR model solution, it is natural to wonder if the exact SIR solution would give an even better description of the data. So in Figs. 16 and 17 we compare data with exact SIR solutions given by the equations of the previous sections. We know from the last section that there is a linear relationship between the asymmetry of data and the basic reproduction number, $\rho$. This has allowed us to obtain an approximate value of $\rho$ and then we have fitted the value of $\beta$ and a normalization factor to the width and height of the data, respectively, and we have added a shift in time to get the position of the peak. The parameters $\rho$ and $\beta$ are given in Table 2. In Fig. 17 we have fitted the US data only to where the first peak is clearly seen. For this reason the other two countries, Brazil and India have not been fitted.

In Figs. 16 and 17, we also plot the results of the Gumbel distribution, but this time the parameters have not been fitted to the data, but to the respective SIR solution. Note that, from Eqs. (43) and (44) that

70 $$\begin{aligned} \beta b= \frac{\ln \rho }{\rho -1-\ln \rho } \end{aligned}$$

From inspections of the numbers given Table 2, columns 4, 5 and 7, we see that this equation is approximately verified. For instance, for $\rho =7$ (Spain and UK), the right-hand side of Eq. (70) gives 0.48, and $1/\beta \simeq 2b$ is roughly verified from Table 2. For $\rho =3$ the equation gives $1/\beta \simeq b / 1.2$ and this is also verified from Table 2 for the case of Belgium.

We see that in general the fit of the Gumbel distribution to the SIR solutions in these countries is good, although it begins to fail, for high values of $\rho$, in the tail part, as we have already seen in the previous section.

Note in Fig. 17 that the USA data are described with the SIR model up to day 150 and cannot be described further because the second wave begins almost immediately. The Gumbel distribution, as we have already mentioned, fails to describe the tail of the SIR solution as a result of the least squares fit, which tends to fit best in the region around the maximum.

From Figs. 16 and 17 we conclude that the data of the countries considered are globally well described with an exact SIR solution, without the need for any time dependence of the parameters. Again we underline that this only happens in the first wave of the countries that we have considered here and not in the other countries or in the remaining waves. The cause of this requires a detailed study of the epidemiological causes that is beyond the scope of this paper.

**Fig. 18:**
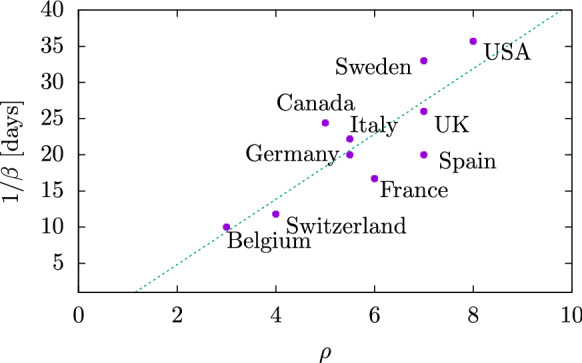
Locations of the different countries studied in the plane of SIR parameters $(\rho , 1/\beta )$

Finally, in Fig. 18 we plot the values of the parameters of the SIR solution in the $(\rho , 1/\beta )$ plane. If we fit a right line to these data, we see that the general trend seems to be for $1/\beta$ to increase with $\rho$. If we exclude the ’outsider’ countries, Belgium and Switzerland (which have small values of $\rho = 3$ or 4) and Sweden and USA (with large values of $\rho =7$, 8), we are left with six countries with similar parameters, in the central zone, around $\rho =6$ and $1/\beta = 20$ d —Spain, France, Italy, UK, Canada and Germany. In these countries a relationship between $\beta$ and $\rho$ is not found. On the other hand, the fact that $\beta$ is similar in these six countries indicates that the probability of recovery is similar in all of them. The recovery probability $\beta$ for an individual should in principle be independent of location. But if we consider that there could be important effects due to medical treatments and hospital capacity to treat severe cases in different countries, this could explain the differences between the $\beta$ values.

Note that in most countries the value of $\rho$ exceeds five, indicating an explosive increase in the first stage of the epidemic in each country. An important consequence of the SIR equations for these high values of $\rho$ is that the total number of people infected during the epidemic reaches almost 100%. Indeed, this is mathematically given by the value of the proper time $\tau$ at the end of the epidemic, which is the solution of Eq. (16). Numerically it is easy to verify that this end value is practically one, for $\rho > 5$. This can also be seen in the lower panel of Fig. 7, where the value of $\tau (t)=r(t)$ for large *t* is practically one. As a consequence, our results indicate that the data from these countries where $\rho >5$ are compatible with an epidemic where practically all initially susceptible individuals were infected, according to the SIR model.

At this point we can already link with the question raised in the introduction of this work, which is whether the lock-downs and other restrictions over the population had any effect in reducing mortality. According to the meta-data study of Ref. [42], the NPI had practically no effect on mortality. This seems to be corroborated in our study for three reasons: (i) that the data are compatible with SIR solutions with high value (${{\mathcal {R}}}_0=\rho \simeq 6$) of the basic reproduction number, which implies that all the susceptible individuals were infected; (ii) that the SIR parameters do not depend on time, but if there were some NPI effects the parameters should be time dependent and the epidemiological curves should differ from the SIR solutions; (iii) there does not seem to be a relationship between the intensity of the lock-down measures and the basic reproduction number. For example, Spain, which had very harsh restrictions, is fitted with the same reproduction number as Sweden, which practically did not have, and is greater than Italy, where the measures were introduced a week earlier.

## Conclusions

In this work we have systematically studied the data from the COVID-19 pandemic using the simplest epidemiological model, the SIR model. With the data from the first wave already consolidated and with a perspective of more than two years, we are in a position to analyze the time series of daily mortality data, verify the validity of the SIR model in this pandemic and extract the parameters of the different countries. The SIR model describes the evolution of an epidemic based solely on statistical laws of proportionality in a sample, and with sufficient data the curve of infected follows a precise and characteristic mathematical law—in fact, in the countries studied we have seen that it describes the data quite accurately.

In the first part of this work we have reviewed the mathematical formalism of the SIR model. First we have solved the differential equations of the SIR model in a parametric way using the proper time as a parameter, defined as the relative number of recovered individuals $\tau =r(t)$. As a function of $\tau$, the SIR solution is analytical, which allows us to study some of its properties, such as, for example, calculating the maximum number of infected $i_\text {peak}$ and the asymptotic number of recovered at the end of the epidemic $r(\infty )$.

Secondly, we have studied the possibility of approximating the SIR solutions using Gumbel distributions *g*(*t*), because this family of functions presents a similar asymmetry as the SIR solutions, and only depends on one parameter, plus the normalization and the position. We have proposed various methods of fitting Gumbel distributions to exact SIR solutions. In particular, using the proper time, we have found simple relationships between the Gumbel parameters and the SIR model parameters.

Third, we have discussed the scaling properties of the exact SIR solutions when plotted as a function of $\beta t$, where $\beta$ is the probability of removal per unit of time. Next we have defined an asymmetry parameter, as the ratio between the right and left half-widths at half-height, of the SIR solutions. We have shown numerically that the asymmetry ${{\mathcal {A}}}(\rho )$ grows almost linearly with the reproduction number $\rho$ and that it is independent of $\beta$. The asymmetry uniquely characterizes the value of $\rho$ and vice versa. Therefore, a measure of the asymmetry of some data of *i*(*t*) at the middle of the height allows to extract the value of $\rho$.

In the results section we have applied the SIR model and the Gumbel distribution to study the daily death-data in the first wave of the COVID-19 pandemic in a dozen countries. The countries have been selected because they are the only ones that present a peak that closely resembles a SIR solution. The data from Spain, France, Italy, UK, Canada, Germany, Belgium, Switzerland and Sweden can be fitted quite well with a SIR solution and also with a Gumbel function. Except for Belgium and Switzerland, data in the rest of the countries are compatible a reproduction number $\rho >5$. This seem to indicate that in practically all susceptible individuals were infected and eventually recovered. This raises questions about the effectiveness of non-pharmaceutical interventions, such as lock-downs, in many countries.

In short, the success of the SIR model to describe the first wave of the COVID-19 pandemic in the countries analyzed has not only allowed us to extract the two parameters that govern the temporal evolution, the basic reproduction number and the constant removal rate, $\beta$. It has also made it possible to carry out a comparative study between the different affected countries.

## Data Availability

The datasets analyzed during the current study are available in the “ourworldindata” repository, https://ourworldindata.org/coronavirus.
